# A New Statistical Modelling Approach to Explain Willingness-to-Try Seafood Byproducts Using Elicited Emotions

**DOI:** 10.3390/foods14152676

**Published:** 2025-07-30

**Authors:** Silvia Murillo, Ryan Ardoin, Bin Li, Witoon Prinyawiwatkul

**Affiliations:** 1 School of Nutrition and Food Sciences, Louisiana State University, Agricultural Center, Baton Rouge, LA 70803, USA; smurillomiguez1@lsu.edu; 2 Food Processing and Sensory Quality Research Unit, Southern Regional Research Center, United States Department of Agriculture-Agricultural Research Service, New Orleans, LA 70124, USA; 3 Department of Experimental Statistics, Louisiana State University Agricultural Center, Baton Rouge, LA 70803, USA; bli@lsu.edu

**Keywords:** seafood processing waste, consumer perception, trial intent, variable selection, safety claim, health claim, sociocultural impact

## Abstract

Seafood processing byproducts (SB) such as bones and skin can be safely used as food ingredients to increase profitability for the seafood sector and provide nutritional value. An online survey of 716 US adult seafood consumers was conducted to explore SB trial intent, responsiveness to health and safety information, and associated elicited emotions (nine-point Likert scale). Consumers’ SB-elicited emotions were defined as those changing in reported intensity (from a baseline condition) after the delivery of SB-related information (dependent *t*-tests). As criteria for practical significance, a raw mean difference of >0.2 units was used, and Cohen’s *d* values were used to classify effect sizes as small, medium, or large. Differences in willingness-to-try, responsiveness to safety and health information, and SB-elicited emotions were found based on self-reported gender and race, with males and Hispanics expressing more openness to SB consumption. SB-elicited emotions were then used to model consumers’ willingness-to-try foods containing SB via logistic regression modeling. Traditional stepwise variable selection was compared to variable selection using raw mean difference > 0.2 units and Cohen’s *d* > 0.50 constraints for SB-elicited emotions. Resulting models indicated that extrinsic information considered at the point of decision-making determined which emotions were relevant to the response. These new approaches yielded models with increased Akaike Information Criterion (AIC) values (lower values indicate better model fit) but could provide simpler and more practically meaningful models for understanding which emotions drive consumption decisions.

## 1. Introduction

Seafood processing byproducts (SB) have been defined as the total of edible and inedible leftovers resulting from production of the main product [1]. These can include bones, skin, scales, shells, viscera, etc. Post-harvest seafood processing often involves several steps to produce the final products that consumers desire (e.g., fish fillets), which can result in substantial byproduct generation—up to over 60% of original biomass (e.g., catfish) [2], depending on the type of seafood.

SB can contain valuable components such as proteins and bioactive peptides, vitamins, collagen, chitin, polyunsaturated fatty acids, and minerals that can be transformed into value-added products [3,4]. Globally, diverse nutritional and functional food applications of SB have been explored, including the addition of collagen from Java barb (fish) skin to yogurt in Indonesia, the use of astaxanthin from shrimp and crab shells as food colorant in Mexico, and the development of a seasoning sauce from anchovy byproducts in South Korea [5], just to name a few. Developing acceptable new foods using edible portions of SB and enhancing their consumption presents an opportunity to reduce waste, contribute to more sustainable food systems, and improve nutrition for a growing global population [3,6]. SB have been more commonly used for food in Asian countries than in the West [7], where the adoption of valorized SB and other upcycled foods faces challenges of consumer acceptance [4].

In addition to consumers’ sociodemographic and psychographic characteristics [8], consumers’ emotions play an important role in food choice in general and related to SB [9]. Consumers’ food-elicited emotions are commonly measured by rating feelings from food-specific lexicons (e.g., the EsSense Profile™ [10]) on a five-point intensity scale or using a check-all-that-apply/rate-all-that-apply format [11]. These types of data have been used to predict or explain food choices using regression modeling [9]. The present study employed a nine-point Likert scale, for increased sensitivity, to evaluate consumer emotions elicited by the concept of SB consumption. In addition, different model selection criteria were evaluated, considering both statistical significance and practical relevance.

The main goals of statistical modeling such as regression analysis are to predict outcomes, explain differences, and/or describe associations between independent variables (predictors or regressors) and dependent variables (responses or outcomes) using empirical data [12]. The decision as to which independent variables form the “best” model depends on the user’s intended purpose [13]. Traditional model selection techniques consider inclusion/exclusion of independent variables based on tests of statistical significance [12]. When comparing food-evoked emotion scores between different conditions or samples, King and Meiselman [11] suggested that mean differences ≤ 0.2 units (on a five-point intensity scale, using the EsSense Profile™) may not be indicative of practical differences, despite showing statistical significance. Therefore, the present study compared logistic regression models built using stepwise backward variable selection [12] with and without the constraint of a >0.2-unit mean difference among conditions (illustrated using the nine-point Likert scale). We also considered the Cohen’s *d* statistic [14], representing a standardized mean difference, as a criterion for independent variable selection in logistic regression models. These methodologies are presented for explanatory (as opposed to predictive) models using SB-elicited emotions.

Along with emotion measurement and modeling strategies, this study also provides updated data on consumers’ responsiveness to SB consumption before and after safety and health information cues. A previous survey focused on product appropriateness and perceived risks associated with SB, while also considering emotions [9]. However, the survey was administered internationally during the COVID-19 pandemic without baseline measurements, which likely impacted reported emotional states. The present survey targeted US consumers’ responses to SB as a concept (rather than product-specific sensory experiences [15,16]) and further evaluated sociocultural impacts of gender and race. The authors propose that properly linking emotions associated with SB to other consumer perceptions can guide both marketing and R&D strategies. To this end, the main objectives of this survey-based research were to evaluate effects of SB—as a concept—on US consumer emotions and to use those emotions in modeling consumers’ trial intent of SB consumption. Using 21 emotions as potential regressors, three variable selection techniques (stepwise backward selection, stepwise with a 0.2-unit constraint, and stepwise with a Cohen’s *d* constraint) were compared to model consumer’s willingness-to-try SB.

## 2. Materials and Methods

### 2.1. Data Collection

A total of N = 716 US consumers participated in a survey to collect data regarding emotions and willingness-to-try SB. The survey was designed using Qualtrics (Qualtrics XM Platform, Provo, UT, USA). Data were collected from May to August 2023.

This research involving human subjects was approved by the Louisiana State University Agricultural Center Institutional Review Board (IRBAG-21-0063; Baton Rouge, LA, USA). Online survey links were distributed by social media/online platforms (e.g., LinkedIn, Facebook), an LSU-Food Innovation Institute consumer database (“Tiger Tasters,” Baton Rouge, LA, USA), and emails. Consumers signed a consent form before starting the survey, and participation was voluntary (no economic reward) and anonymous. Participants who did not meet the following screening criteria were excluded from the study: adults (at least 18 years old), seafood consumers (“Do you consume fish or seafood products?” = yes), and US consumers (“Have you lived in the United States of America for the last three years?” = yes). Demographic (age, gender, race, education level) information was collected.

### 2.2. Questionnaires

Consumers rated 21 emotions selected from the Essence Profile^®^ (*active*, *adventurous*, *aggressive*, *bored*, *calm*, *eager*, *energetic*, *enthusiastic*, *free*, *friendly*, *good*, *glad*, *happy*, *healthy*, *loving*, *nostalgic*, *pleased*, *peaceful*, *satisfied*, *unsafe*, and *worried* [10]) using 9-point Likert scales (disagree extremely = 1, disagree very much = 2, disagree moderately = 3, disagree slightly = 4, neither disagree or agree = 5, agree slightly = 6, agree moderately = 7, agree very much = 8, agree extremely = 9). Terms from the Essence Profile^®^ were chosen for compatibility with US consumers, because it was developed in the US as an English-language emotion lexicon and has been commonly used in Western consumer food research. Specific emotions were selected for this study based on predicted or previously observed relevance to SB [9,15,16], and *healthy* and *unsafe* were added in relation to interventions of SB health benefit and safety information.

First, baseline emotion profiles were obtained. Consumers were asked “How do you feel today?” Prior to rating emotions a second time (in relation to seafood byproduct (SB) consumption), consumers were asked a series of questions related to SB, which are presented in the next paragraph. This section of the questionnaire also provided consumers with a definition of SB. Finally, consumers rated emotions in response to the question, “How does the idea of consuming seafood byproducts make you feel?”

After rating baseline emotions and prior to SB-elicited emotions, consumers were asked the following questions: “Are you typically willing to try new foods?” (yes/not sure/no); “Do you know what food byproduct is?” (yes/no). The following SB definition was then provided: “Related to seafood and aquacultural products, a byproduct is something (for example, bone, skin, gut) which is produced during the manufacture or processing of another product (for example, catfish fillet).” Consumers were then asked, “Have you ever eaten food products that contain or are fortified with seafood or aquacultural (farm-raised) byproducts such as head, bones, gut, or skin?” (yes/maybe/no).

The following questions were answered (yes/maybe/no) sequentially in relation to consumers’ willingness-to-try seafood byproducts: “Would you be willing to try new products containing a small portion of seafood byproducts such as bone and skin?” (general willingness-to-try question; WTT); “Would you be willing to try new products containing a small portion of seafood byproducts such as bone and skin, knowing it is safe for consumption?” (with safety information; WTTS); “Would you be willing to try new products fortified with safe seafood byproducts that are claimed to provide health benefits?” (with safety and health benefit information; WTTSH).

### 2.3. Data Analysis

Dependent *t*-tests were used to analyze SB-elicited emotions (comparing emotion responses to the idea of SB consumption to baseline emotions). Demographics were reported as percentages of the total population sample and used to explore differences in emotions, WTT, WTTS, and WTTSH by race and gender. For willingness-to-try SB questions (WTT, WTTS, and WTTSH), percentages of “yes” responses were reported, and changes in response distributions at each subsequent information level (from WTT to WTTS, and from WTTS to WTTSH) were investigated by Stuart–Maxwell tests followed by McNemar’s tests, which considered only changes in “yes” responses.

Logistic regression analysis was conducted using emotions measured after SB information as regressors and WTT, WTTS, and WTTSH as responses (yes = 1, maybe/no = 0), respectively. Odds ratios estimates were expressed in terms of the probability of a “yes” response in each model. Four variable selection techniques were utilized and compared for each response: 1. a full model including all 21 emotions, interpreting only those with Wald’s *p*-value ≤ 0.05; 2. stepwise backward variable selection [12]; 3. stepwise selection plus a constraint of >0.2-unit mean difference between baseline and SB emotions (“King’s model”) [11]; and 4. stepwise selection plus a constraint of Cohen’s *d* values ≥ 0.5 between baseline and SB emotions (“Cohen’s model”). Cohen’s *d* was calculated as shown in Equation (1). Cohen’s *d* values were also used to define effects sizes as negligible (*d* < 0.2), low (0.2 ≤ *d* < 0.5), medium (0.5 ≤ *d* < 0.8), or high (*d* ≥ 0.8) [14].

Equation (1).

(1) Cohen’s *d* = |(Mean of baseline emotion − Mean of SB-related emotion)|/(Pooled standard deviation)

Data were analyzed by Microsoft^®^ Excel^®^ (Version 2206 Build 16.0.15330.20260; Microsoft Corporation, Redmond, WA, USA) and R software version 4.0.3 (RStudio, Inc., Boston, MA, USA), with α = 0.05 significance level used throughout.

## 3. Results and Discussion

### 3.1. Emotions Elicited by Seafood Byproducts

By first measuring consumers’ emotions at the beginning of the survey then immediately after invoking SB information, SB-related emotions were revealed as those shifting from the baseline condition (Table 1). Significant differences were observed in 18 of 21 emotions measured, with 16 positive valence emotion scores decreasing and negative emotions *aggressive* and *unsafe* increasing in magnitude (based on dependent *t*-tests). Positive emotion ratings were suppressed, such that none exceeded a mean value of 6 (“slightly agree”). In previous studies, mostly positive emotions were associated with acceptable foods made with catfish bone powder [15] and skins [16] after blind tasting and subsequent safety and/or health information. Collectively, these results suggest that favorable sensory experiences may be sufficient to override potentially negative preconceptions of SB consumption and enhance the impact of benefit information, but this hypothesis was not formally tested via the online survey.

Despite providing informational cues related to the safety and health benefits of seafood byproducts, mean *healthy* scores significantly dropped, and *unsafe* scores significantly increased (*t*-tests; Table 1). Using the Cohen’s *d* statistic as a follow-up estimate of practical significance permitted categorization of SB-elicited emotions by effect size, with *unsafe* exhibiting a large effect (d = 0.8) and *healthy* exhibiting a medium effect (*d* = 0.5). Still, an SB safety message had a positive influence on consumers’ willingness-to-try foods made with SB (discussed later), potentially mitigating perceived feelings of *unsafe*.

Other large effect sizes were found for reductions in *calm, friendly*, *good*, *happy*, and *loving*. Comparing statistical differences in emotion means (*t*-test), raw mean differences > 0.2 units, and standardized effect sizes (Choen’s *d* ≥ 0.5), only one discrepancy was found. *Worried* scores were not statistically different at α = 0.05, and the effect size was negligible (*d* = 0.1), but the raw mean difference between conditions exceeded 0.2 (mean difference = 0.28; Table 1)—demonstrating the rigor added by considerations of observed variance. For further resolution, consumer segmentation was explored (see Section 3.2).

### 3.2. Differences in Emotional Profiles by Gender and Race

Comparing male and female baseline emotion ratings, males began the survey reporting statistically higher baseline levels of *aggressive* and *energetic*, while females scored higher in *loving* and *worried* (Table 2). No other significant baseline differences were observed. In relation to SB consumption however, males reported significantly higher mean scores across emotions except for *worried* (higher in females) and *unsafe* (no significant difference), albeit with small effect sizes. While these results suggest that males may respond more favorably to SB consumption, a similar trend of decreasing positive emotions was observed for both genders in response to SB. The observed emotional differences may have also been influenced by broader personality predispositions, where females have shown to react in response to anxiety sensitivity (e.g., more *worried*) and males in relation to the sensation-seeking trait (e.g., more *adventurous*, *eager*, and *energetic*) [17].

US consumers who participated in the study were asked to self-report their race, which, for the purposes of this study, will be interpreted as an indicator of sociocultural identity rather than biological information [18]. Differences in the baseline emotional profiles of White and Hispanic participants only indicated significant differences in *enthusiastic* and *unsafe* (both higher in Hispanic consumers; Table 3). Race/ethnicity differences in regulating and reporting emotions have been seen elsewhere [19], and in the present study, Hispanic consumers reported significantly higher levels of 16 positive emotions related to the idea of SB consumption than white participants. Previous studies of SB have also evidenced this sociocultural difference [15], where Latin American consumers presented higher odds of purchasing products made with catfish bone powder. The design of the present study, using baseline emotions as a “control” condition, allowed for the isolation of SB-related effects as well as differences in sociocultural reactions to SB.

### 3.3. Effects of Safety and Health Informational Cues on Willingness-to-Try Seafood Byproducts

**Figure 1:**
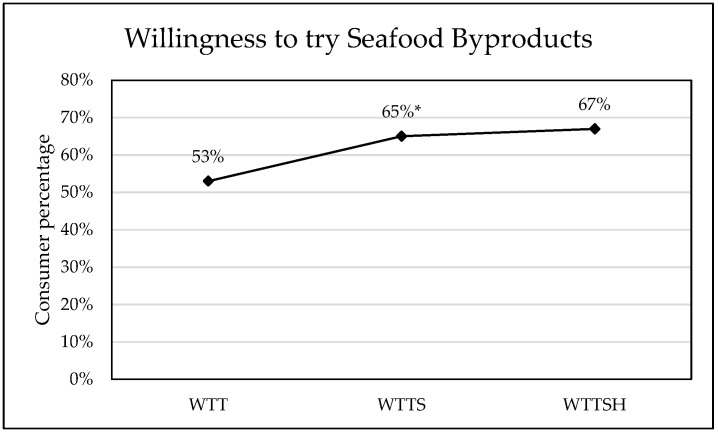
Percentage of consumers’ willing-to-try seafood byproducts (“yes” responses) under different information conditions: willingness-to-try (WTT), willingness-to-try with safety information (WTTS), and willingness-to-try with safety and health benefit information (WTTSH). * Significant increase in “yes” responses (McNemar’s test, α = 0.05) with additional information from one condition to the next (*p*-value < 0.05).

SB safety information positively impacted consumers’ willingness-to-try new products containing a small portion of SB. Over half of the current 716 respondents (53%) expressed positive willingness-to-try SB after being provided with a definition only (WTT = “yes”; Figure 1). In a previous survey of SB perceptions distributed internationally during the COVID-19 pandemic, 47% of participants responded affirmatively to the WTT question [9]. Based on previous research [16], it was hypothesized that sequential informational cues (i.e., WTTS followed by WTTSH) would exhibit an additive effect on the proportion of “yes” responses for SB trial intent. However, only the safety message had a significantly positive effect on US consumers’ openness to SB, boosting the percentage of WTTS = “yes” responses to 65% (Figure 1). An additional safety and health message further increased willingness directionally (67% WTTSH; Figure 1) but without significant impact (Table A1).

For SB, sensory quality and safety risks have been considered barriers to SB consumption [9]. Similarly, in Spain, negative opinions of meat byproducts were linked to poor sensory attributes as well as safety concerns over chemicals such as toxins or drug residues [20]. Hellai et al. [21] found that the provision of positive (environmental or health-related) information increased risk-averse Canadians’ willingness to pay for upcycled products, and vice versa for negative information with risk-tolerant consumers. In light of the positive influence of safety information on US consumers’ SB trial intent (Figure 1) alongside the elicitation of *unsafe* emotions (Table 1), subjective ambivalence should be considered. Subjective ambivalence arises from product evaluations where both risks and benefits are considered, and it is characteristic of consumers’ behavioral intentions toward byproducts. Subjective ambivalence among European consumers explained reduced purchase intent of novel bio-based products, beyond emotions alone [22]. To alleviate ambivalence, individuals select sources of information which favor one direction or the other [22]. Therefore, while extrinsic product information has been suggested to promote acceptance of unfamiliar foods [20,21] as in the present study, consumers’ decision-making regarding SB consumption may be influenced holistically by sensory properties, safety, nutrition, and emotions.

### 3.4. Willingness-to-Try Seafood Byproducts by Gender and Race

**Figure 2:**
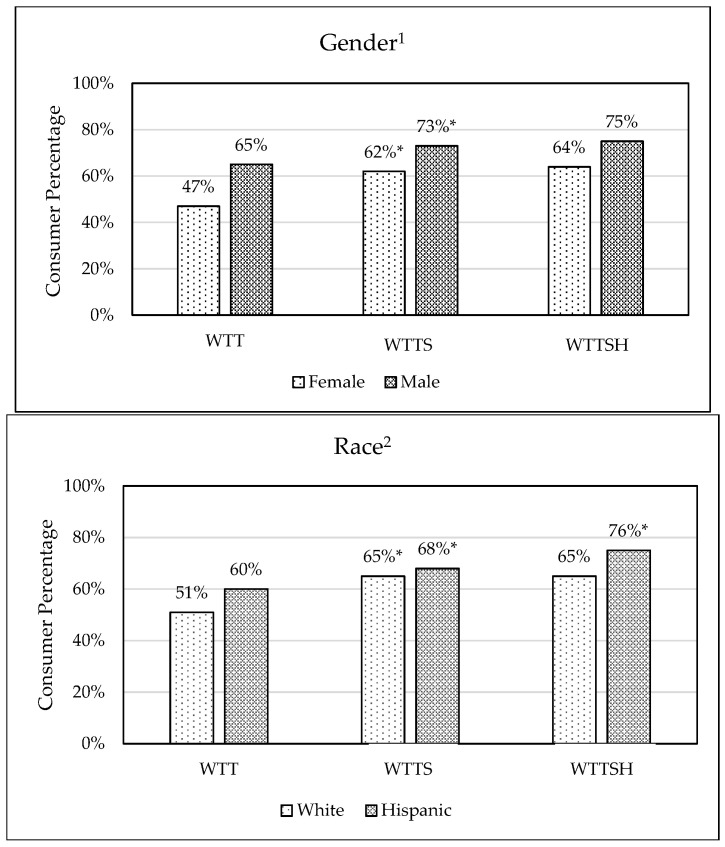
Percentage willing-to-try seafood byproducts (“yes” responses), by gender^1^ and race^2^, under different information conditions: willingness-to-try (WTT), willingness-to-try with safety information (WTTS), and willingness-to-try with safety and health benefit information (WTTSH). * There was a significant increase in “yes” responses (McNemar’s test) with additional information from WTT to WTTS (*p* < 0.001) and from WTTS to WTTSH (*p* < 0.01).

A higher proportion of male versus female consumers were willing to try foods containing SB. This was true under all information conditions (WTT, WTTS, and WTTSH; Figure 2) and is typical with unusual ingredients, such as edible insects, in Western cultures [23,24]. This trend has been associated with higher rates of food neophobia and disgust sensitivity in women [17,23,25]. However, the largest increase in “yes” responses, from 47% to 62%, was presently observed for females after SB safety and health information.

In survey-based designs, expression of gender differences can depend on culture and phrasing of questions and messaging. Rather than “byproducts,” other studies have described related concepts of “waste-to-value,” “circular economy,” and “upcycling” [26,27]. A survey of 477 Italian consumers found that males were less likely to buy waste-to-value products [26]. In other research, females responded more favorably to environmental appeals for upcycled food [28]. Regarding SB, the current findings aligned with our previous analysis [9] where males had greater odds of SB trial intent.

Along with the breadth of other factors influencing food choice, sociodemographic features have shown associations with consumers’ attitudes and acceptance of byproducts [8,9]. Dietary habits are often associated with deep-rooted customs, and increasing racial diversity is projected to shape food demand in the US [29]. Racial information, as a social dimension, can add explanatory power to measures of behavioral preferences [30].

From data collected during the COVID-19 pandemic, White consumers were less willing to try SB than Hispanic/Latin/Spanish origin consumers (>15% difference). From this more recent dataset (Figure 2), Hispanic consumers were still more open to SB consumption and showed greater responsiveness to informational cues. Both White and Hispanic respondents demonstrated a significant increase from WTT to WTTS responses (increases of 14% and 8%, respectively; Figure 2). While no effect was seen in WTTSH for White consumers, Hispanics’ “yes” response frequency increased from 68% to 76%. Other research has pointed to Hispanic/Latino consumers as one of the most willing ethnic groups to try novel food sources [15], and this growing US demographic may encompass potential early adopters of SB.

### 3.5. Emotions Explaining Consumers’ Willingness-to-Try Seafood Byproducts

#### 3.5.1. The Willing Versus Reluctant Approach

**Figure 3:**
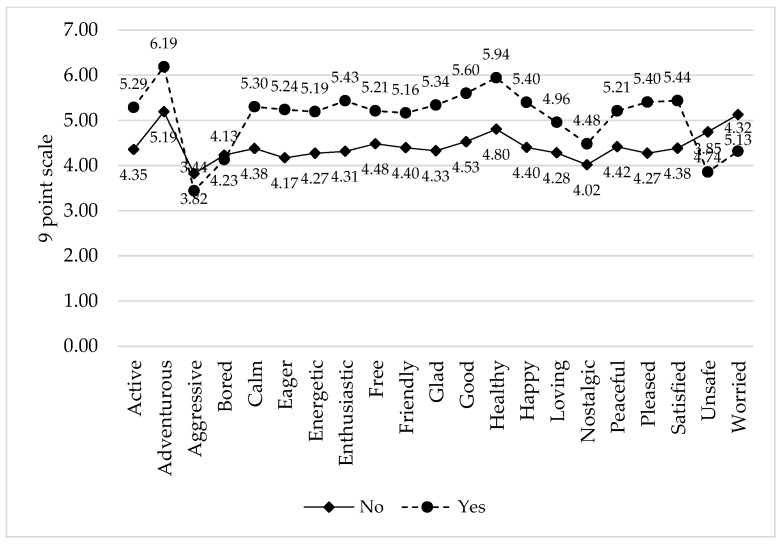
Mean emotion intensities (9-point scale) elicited by consumers regarding seafood byproducts, separated by willingness-to-try foods made with seafood byproducts after safety and health benefit information.

To further illustrate the (often latent) relationships between emotions and food choice, participants in the present survey were segmented based on their response to the WTTSH question. Mean emotion scores for “willing” (WTTSH = “yes”) and “reluctant” (WTTSH = “maybe” or “no”) consumers of SB were compared (Figure 3). These results clearly indicated a divergence in emotion responses between the groups, with willing consumers reporting higher levels of positive emotions, and reluctant consumers reporting higher levels of negative emotions (except for *bored*; Figure 3). Notably, these mean differences exceeded the 0.2-unit criterion for practical difference suggested by King et al. [11], which was employed in logistic modeling (see next section of this manuscript).

#### 3.5.2. Selection of Emotion Variables in Logistic Regression Models Under Different Information Conditions

This study presents a new approach to selection of emotion variables in logistic regression (logit) models using established heuristics to assign practical importance. These models start with statistically significant regressors, then impose additional constraints based on raw mean difference [11] and standardized mean difference [14] from a baseline condition. Thus, in exploring this approach, simplified models were obtained using only emotions relevant to the product category (seafood byproducts).

The full model, comprising all 21 emotions measured after SB information, produced six significant regressors for WTT and four significant regressors for WTTS and WTTSH (Table 4). Preestablished emotion lexicons seek to represent the full range of potential food-evoked emotions, and this number of candidate variables (e.g., 21 form the EsSense Profile^®^ [10]) can be too numerous for unbiased statistical modeling [12]. Full models can become complex with interpretation of significant regressors based upon dependencies with other independent variables in the model. As such, simpler models are often preferred for interpretability, practical usefulness, and ease of validation [12].

Using stepwise backward variable selection, reduced models identified seven emotions significant to the WTT response and six for WTTSH—more than those indicated from the full model. Specifically, *friendly* and *happy* were included in the reduced WTT model, and *worried* was excluded. The reduced model for WTTSH incorporated *adventurous*, a sensation-seeking emotion which has also been associated elsewhere with SB consumption and other novel foods [16,24]. *Adventurous* was also significant to both full and stepwise models of WTTS. In all cases, AIC values were lower (preferred) for the reduced stepwise model, penalizing overparameterization.

The AIC metric was originally designed for prediction of future outcomes, not for explanatory power per se; the same is true of stepwise variable selection [13]. The focus of present modeling approaches was to explain trial intent toward SB consumption based on practically relevant SB-elicited emotions. Therefore, only emotions with a raw mean difference > 0.2 between baseline and eliciting conditions were retained in the so-called (for the purpose of this manuscript) “King’s model.” For what we are labeling “Cohen’s model,“ emotions with a Cohen’s *d* value ≥ 0.5 indicating a medium effect size were retained from the stepwise reduced model.

In this case, King’s model and Cohen’s model criteria produced the same set of regressors for each respective response (Table 4). WTT models did not deviate from the initial stepwise reduced model. For WTTSH, *adventurous* and *worried* were removed from the stepwise iteration, as they did not meet the stated criteria for practical significance to SB. The remaining emotions were all clearly positively valanced. Notably, the King’s and Cohen’s models of WTTS retained only the emotion *pleased*. This extreme of model reduction to a single regressor may sacrifice information as indicated by AIC values. In these theoretical explanatory models, a tradeoff was observed between empirical precision and practical interest [13] and between information and parsimony [31].

A key takeaway from this study was that the emotions which significantly explained trial intent of SB depended upon what information consumers were exposed to upon decision making (among WTT, WTTS, and WTTSH scenarios; Table 4). In this regard, SB-related information may be considered “nudges”—subtle interventions which influence consumers’ responses in predictable ways [32]—toward emotions and food choice. In real-life situations, this effect may translate to extrinsic cues influencing emotions at the point of purchase decision [33]. From a research perspective, investigating information cues as nudges may guide SB R&D or marketing efforts beyond intrinsic sensory properties, or target emotions which favor healthy or sustainable consumption decisions. Factors such as the context and placement of SB information should be considered for practical applicability.

### 3.6. Limitaions

A goal of this study was to present new variable selection approaches based on previously cited criteria for practical significance [11,14]. However, this study employed a nine-point emotion rating scale instead of a five-point scale from which the 0.20-unit mean difference criteria was derived (for King’s model [11]). As no other numerical cut-off has been proposed for food-evoked emotions, a 0.2-unit mean difference was used to demonstrate this new statistical modeling approach, rather than for interpretation. While a multiplier of 1.8 would provide a relative adjustment from a five-point to nine-point scale, yielding a mean difference criteria of 0.36 units (1.8 × 0.2 = 0.36), future research is needed to validate such a conversion based on scale utilization.

Since Cohen’s *d* statistic represents a standardized mean difference, scale size is less relevant. The conventional benchmark for a medium effect size was used for consideration of practical differences (for Cohen’s model), but in some cases, researchers may favor a less stringent criteria to include potentially small but relevant changes in consumer emotions. These considerations depict the inherent tradeoff between model information/complexity and useability/simplicity, which can be tailored to the researcher’s purpose [10,11].

One inherent limitation to the online survey-based methods was self-selection bias [34], which limits the generalizability of results. Responses were collected via voluntary participation from specific online recruitment channels (e.g., Facebook, LinkedIn), which necessarily excluded non-internet users and included only interested participants exposed to recruitment posts on these platforms. Secondly, self-reporting in survey questionnaires may favor socially desirable or normative responses [35] and do not necessarily translate to behavioral outcomes. Additionally, consumers’ responses were based on subjective constructs of SB rather than exposure to actual products. Therefore, future research should seek to validate the presently observed results with target consumer segments and real products.

## 4. Conclusions

Separate information cues regarding seafood byproduct (SB) safety and potential health benefits dictated which emotions were significant in explaining US consumers’ trial intent. Safety information effectively increased consumers’ willingness-to-try foods containing SB, but the idea of SB consumption elicited decreased positive emotions and increased *unsafe* feelings among consumers. From these data, new approaches to food-evoked emotion measurement and modeling included the use of a nine-point Likert scale, assigning practical importance (beyond statistical significance) to SB-elicited emotions, and comparing logistic regression models with practically important SB-elicited regressors to traditional model selection. By modeling willingness-to-try with only practically relevant emotions, simpler models were obtained at the expense of larger AIC values. Variable selection constraints of raw mean difference (from baseline emotions) > 0.2 and medium-or-larger effect sizes (Cohen *d* ≥ 0.5) produced equivalent models under all information conditions. However, these criteria may be adjusted based on pragmatic considerations (e.g., five-point vs. nine-point scale), research purpose (explaining vs. predicting), or background knowledge of the target population. From the present survey, males and Hispanic consumers were most open to seafood byproduct trial, exhibiting more favorable emotion profiles.

## Figures and Tables

**Table 1:** Mean emotion intensities (9-point scales) from baseline condition and those elicited by seafood byproduct consumption.

Emotions	Baseline	Seafood Byproducts	Cohen’s *d*	Mean Difference ^^^
Active	6.07	4.98 *	0.6 ^m^	**1.09**
Adventurous	5.87	5.86	0.1 ^n^	0.01
Aggressive	2.84	3.57 *	0.4 ^s^	**0.73**
Bored	4.32	4.16	0.1 ^n^	0.15
Calm	6.45	5.00 *	0.9 ^l^	**1.45**
Eager	5.90	4.89 *	0.6 ^m^	**1.01**
Energetic	5.79	4.89 *	0.5 ^m^	**0.9**
Enthusiastic	6.09	5.07 *	0.6 ^m^	**1.02**
Free	6.24	4.97 *	0.7 ^m^	**1.27**
Friendly	7.06	4.91 *	1.4 ^l^	**2.15**
Glad	6.50	5.01 *	0.9 ^l^	**1.49**
Good	6.86	5.25 *	1.0 ^l^	**1.62**
Healthy	6.46	5.57 *	0.5 ^m^	**0.89**
Happy	6.68	5.07 *	1.0 ^l^	**1.6**
Loving	6.89	4.74 *	1.3 ^l^	**2.15**
Nostalgic	5.35	4.33 *	0.6 ^m^	**1.03**
Peaceful	6.47	4.95 *	0.9 ^l^	**1.52**
Pleased	6.33	5.03 *	0.8 ^l^	**1.3**
Satisfied	6.37	5.09 *	0.8 ^m^	**1.28**
Unsafe	2.62	4.15 *	0.8 ^l^	**1.53**
Worried	4.86	4.58	0.1 ^n^	0.28

* Significantly different between conditions based on dependent *t*-test (α = 0.05); ^n,s,m,l^ Cohen’s *d* values defined as n = negligible (*d* < 0.2), s = small (0.2 ≤ *d* < 0.5), m = medium (0.5 ≤ *d* < 0.8), or l = large (*d* ≥ 0.8) effect size between conditions; ^^^ Bold values represent a practical mean difference of >0.2 units between conditions.

**Table 2:** Comparing mean emotion intensities (9-point scales) between genders from baseline condition and those elicited by seafood byproducts consumption.

	Baseline		Seafood Byproducts	
Emotions	Female	Male	Female	Male
Active	5.98	**6.26**	4.85	5.26 *^,s^
Adventurous	5.79	**6.04**	5.78	6.04 *
Aggressive	2.67	3.21 *^,s^	3.45	3.82 *^,s^
Bored	4.34	4.27	4.04	4.43 *^,s^
Calm	6.41	6.56	4.9	5.21 *
Eager	5.87	5.96	4.75	5.19 *^,s^
Energetic	5.67	6.05 *^,^ ^n^	4.73	5.24 *^,s^
Enthusiastic	6.05	6.18	4.95	5.33 *^,s^
Free	6.24	6.22	4.83	5.28 *^,s^
Friendly	7.12	6.94	4.8	5.15 *^,s^
Glad	6.51	6.48	4.92	5.21 *
Good	6.91	6.76	5.15	5.46 *^,s^
Healthy	6.44	6.51	5.48	5.76 *
Happy	6.71	6.6	4.94	5.37 *^,s^
Loving	6.99	6.66 *^,^ ^n^	4.63	4.97 *^,s^
Nostalgic	5.35	5.35	4.24	4.53 *
Peaceful	6.43	6.57	4.83	5.22 *^,s^
Pleased	6.31	6.39	4.91	5.30 *^,s^
Satisfied	6.37	6.36	4.97	5.37 *^,s^
Unsafe	2.62	2.63	4.17	4.09
Worried	5.05	4.46 *^,s^	4.73	4.26 *^,s^

* Significantly different between genders within a respective condition based on independent *t*-test (α = 0.05); ^n,s^ Cohen’s *d* values defined as n = negligible (d < 0.2), s = small (0.2 ≤ d < 0.5) effect size between conditions; Bold values represent a practical mean difference of > 0.2 between genders within a respective condition.

**Table 3:** Comparing mean emotion intensities (9-point scales) between White and Hispanic consumers from baseline condition and those elicited by seafood byproducts’ consumption.

	Baseline		Seafood Byproducts	
Emotions	White	Hispanic	White	Hispanic
Active	5.99	6.28	4.79	5.25 *^,s^
Adventurous	5.87	5.92	5.75	6.24 *^,s^
Aggressive	2.76	2.65	3.57	3.34
Bored	4.25	4.24	4.14	4.34
Calm	6.4	6.43	4.79	5.33 *^,s^
Eager	5.85	5.97	4.69	5.15 *^,s^
Energetic	5.72	6.02	4.7	5.40 *^,s^
Enthusiastic	6.01	6.43 *	4.85	5.59 *^,s^
Free	6.24	6.37	4.77	5.35 *^,s^
Friendly	7.06	6.9	4.76	5.24 *^,s^
Glad	6.48	6.6	4.83	5.42 *^,s^
Good	6.85	6.79	5.03	5.70 *^,s^
Healthy	6.42	6.56	5.4	6.10 *^,s^
Happy	6.71	6.63	4.89	5.46 *^,s^
Loving	6.83	6.89	4.54	5.11 *^,s^
Nostalgic	5.33	5.44	4.22	4.30
Peaceful	6.44	6.35	4.74	5.34 *^,s^
Pleased	6.32	6.51	4.86	5.40 *^,s^
Satisfied	6.36	6.39	4.94	5.46 *^,s^
Unsafe	2.38	3.08 *	4.13	4.25
Worried	4.77	4.99	4.59	4.70

* Significantly different between races within a respective condition based on independent *t*-test (α = 0.05); s Cohen’s *d* values defined as s = small (0.2 ≤ *d* < 0.5) effect size between conditions; Bold values represent a practical mean difference of >0.2 between races within a respective condition.

**Table 4:** Logistic regression model selection ^1^ of US consumers’ willingness-to-try seafood byproducts under different information conditions ^2^.

	WTT		WTTS		WTTSH	
	Emotions	OR ^3^	Emotions	OR	Emotions	OR
Full Model (21 emotions) AIC WTT = 871.41 AIC WTTS = 808.26 AIC WTTSH = 784.04	Eager	1.25	Adventurous	1.23	Eager	1.25
	Free	0.75	Eager	1.21	Healthy	1.26
	Good	1.27	Pleased	1.32	Satisfied	1.33
	Satisfied	1.32	Worried	0.78	Worried	0.79
	Unsafe	0.85				
	Worried	0.87				
Stepwise Model AIC WTT = 855.06 AIC WTTS = 796.12 AIC WTTSH = 767.08	Eager	1.29	Adventurous	1.30	Adventurous	1.16
	Free	0.76	Pleased	1.54	Eager	1.30
	Friendly	0.80	Worried	0.73	Healthy	1.33
	Good	1.31			Loving	0.77
	Happy	1.29			Satisfied	1.43
	Satisfied	1.36			Worried	0.73
	Unsafe	0.77				
King’s model AIC WTT = 855.06 AIC WTTS = 832.56 AIC WTTSH = 818.75	Eager	1.29	Pleased	1.78	Eager	1.33
	Free	0.76			Healthy	1.46
	Friendly	0.80			Loving	0.77
	Good	1.31			Satisfied	1.42
	Happy	1.29				
	Satisfied	1.36				
	Unsafe	0.77				
Cohen’s d Model AIC WTT = 855.06 AIC WTTS = 832.56 AIC WTTSH = 818.75	Eager	1.29	Pleased	1.78	Eager	1.33
	Free	0.76			Healthy	1.46
	Friendly	0.80			Loving	0.77
	Good	1.31			Satisfied	1.42
	Happy	1.29				
	Satisfied	1.36				
	Unsafe	0.77				

^1^ Full model included all 21 emotions, showing only those with Wald’s *p*-value ≤ 0.05; stepwise model used backward stepwise variable selection; King’s model used stepwise selection plus a constraint of > 0.2-unit mean difference from baseline emotions; and Cohen’s model used stepwise selection plus a constraint of Cohen’s *d* values ≥ 0.5 compared between baseline and SB emotions. ^2^ N = 716 consumers reported willingness-to-try (WTT), willingness-to-try with safety information (WTTS), and willingness-to-try with safety and health benefit information (WTTSH). ^3^ Odds ratios estimates expressed in terms of a “yes” response. Emotions were rated on a 9-point scale.

## Data Availability

The original contributions presented in this study are included in the article. Further inquiries can be directed to the corresponding author.

## Appendix A

### Appendix A.1

**Table A1:** Comparing consumers’ willingness-to-try seafood byproducts under different information conditions ^1^ for gender.

McNemar Test Result	WTT vs. WTTS	WTTS vs. WTTSH
Chi-square statistic	71.05	1.61
*p*-value	<0.001	0.2046

^1^ Based on “yes” responses for willingness-to-try (WTT), willingness-to-try with safety information (WTTS), and willingness-to-try with safety and health benefit information (WTTSH).
